# Reflex Modification Audiometry Reveals Dual Roles for Olivocochlear Neurotransmission

**DOI:** 10.3389/fncel.2017.00361

**Published:** 2017-11-22

**Authors:** Paul D. Allen, Anne E. Luebke

**Affiliations:** ^1^Department of Otolaryngology, University of Rochester Medical Center, Rochester, NY, United States; ^2^Department of Neuroscience and the Ernst J. Del Monte Institute for Neuroscience, University of Rochester Medical Center, Rochester, NY, United States; ^3^Department of Biomedical Engineering, University of Rochester, Rochester, NY, United States

**Keywords:** calcitonin gene-related peptide (CGRP), alpha 9 nicotinic acetylcholine receptor, Ld'T alpha 9 nicotinic acetylcholine receptor, lateral olivocochlear efferent (LOC), medial olivocochlear efferent (MOC), hearing in noise, prepulse inhibition, acoustic startle

## Abstract

Approximately 15% of American adults report some degree of difficulty hearing in a noisy environment or have auditory filtering difficulties. There are objective clinical tests of auditory filtering, yet few tests exist for mouse models that do not rely on extensive training. We have used reflex modification audiometry (RMA) and developed exclusion criteria for the mouse model. This RMA based test makes use of the acoustic startle response (ASR) and the ability of prepulses to inhibit the ASR [i.e., prepulse inhibition (PPI)] to assess the mouse's ability to detect prepulse signals presented in quiet or embedded in masking noise. We have studied PPI behavior across four inbred mouse strains with normal cochlear function and developed pre-testing exclusion criteria and test/retest reliability measures. Moreover, because both the medial (MOC) and the lateral (LOC) olivocochlear efferent feedback systems have been proposed to improve auditory behavior performance, especially in noisy backgrounds, we have examined PPI abilities in mice (with their littermate controls) either lacking the MOC receptor subunit α9 nicotinic acetylcholine receptor [α9 nAChR (–/–)] or expressing an overactive receptor [Ld'T mutation in α9 nAChR KI], or lacking an LOC efferent neuropeptide, alpha calcitonin gene-related peptide [αCGRP (–/–)] only in the CNS. Because CGRP receptor formation has been shown to mature from juvenile to adult ages, we also studied if this maturation would be reflected in PPI behavioral responses in juvenile and adult (+/+) controls and in adult αCGRP (–/–) animals. We show that 50% PPI response thresholds (sound level with 50% correct responses) in quiet are *decreased* in the (–/–) α9 nAChR animals, and 50% PPI responses are *increased* for mice with an overactive receptor (α9 nAChR KI) and are increased in adult mice lacking αCGRP (–/–). However, in background noise, only mice lacking αCGRP exhibited increased 50% PPI response thresholds, as there were no significant differences between α9 nAChR adult mouse lines and their littermate controls. These findings suggest that MOC and LOC olivocochlear neurotransmission work in tandem to improve behavioral responses to sound. These experiments further pave the way for rapid behavioral hearing assessments in other mouse models.

## Introduction

Approximately 15% of American adults report some degree of difficulty hearing in a noisy environment (Fausti et al., 2005; NIDCD, 2010). There are objective tests of hearing-in-noise or auditory filtering abilities for the human population, yet, few such tests exist for mouse models that do not rely on extensive training. Such a lack of hearing-in-noise assessments in the mouse model can inhibit its use to understand the molecular underpinnings that may underlie auditory filtering abilities. Moreover, the olivocochlear (OC) efferent feedback system has been proposed to aid in auditory filtering (i.e., hearing in noise detection), yet due to effects of olivocochlear efferent feedback on aging there is a need for more rapid assessment tools (Lauer and May, 2011; May et al., 2011; Liberman et al., 2014). The OC efferent feedback system is divided into a medial system (MOC) and lateral (LOC) systems. The main neurotransmitter of the MOC system is acetylcholine (ACh) and the MOC projects from the auditory brainstem back to outer hair cells of the cochlea synapsing on nicotinic receptors containing α9 and α10 nicotinic ACh receptor subunits. The lateral system (LOC) contains three neurotransmitters, namely ACh, GABA, and alpha Calcitonin Gene-Related peptide (CGRP), and fibers containing these neurotransmitters project from the auditory brainstem to the cochlear afferent nerve. Loss of either the MOC or LOC system after cochlear development does not affect auditory thresholds, yet both systems are activated by sound. Sound suppresses MOC responses (as assessed by otoacoustic emissions and cochlear action potentials and nerve activity) and CGRP transmission through the LOC system has been shown to enhance suprathreshold cochlear nerve activity suggesting that both systems may work in tandem to enhance behavioral responses to sound (Maison et al., 2002, 2003a; Guinan et al., 2005; Guinan, 2006, 2010, 2013, 2014; Le Prell et al., 2014a,b; Dickerson et al., 2016).

We have used reflex modification audiometry (RMA), which makes use of the acoustic startle response (ASR) and the ability of prepulses to inhibit the ASR [i.e., prepulse inhibition (PPI)] to assess the mouse's ability to detect prepulse signals presented in quiet or embedded in masking noise. RMA has long been used (Hoffman and Ison, 1980; Ison and Hoffman, 1983; Ison et al., 2017), has known physiological bases (Braff et al., 2001; Geyer and Swerdlow, 2001), and has been extended to mouse models (Lobarinas et al., 2013; Longenecker et al., 2016; Lauer et al., 2017). However, we have developed exclusion criteria with embedded test/retest reliability measures. In addition, we use a new metric, the ASR ratio, defined as the startle response in masking noise divided by the response in quiet, to ensure baseline startle is robust in the presence of background masking noise before preceding onto tests of PPI.

Moreover, as the background strains of many transgenic mouse models differ, there is a need for a method to quantitatively assess hearing in noise ability that is effective for many mouse strains. We have begun to study this hearing-in-noise behavior across four inbred mouse strains (CBA/CaJ, C57BL6/J, 129SvEvTac, C57BL6/J × 129SvEvTac). There were no differences in cochlear function in these background strains when assessed (<3 months of age before any age-related hearing loss) using auditory brainstem response thresholds (ABRs) and distortion product otoacoustic emissions (DPOAEs) response magnitudes, yet the various background strains may differ in their auditory behavioral responses (Johnson et al., 1997, 2006; Ingham et al., 2011).

Because both the MOC and the LOC olivocochlear efferent feedback systems have been proposed to improve hearing in background noise, here we test the idea that efferent MOC/LOC work together to improve behavioral responses using RMA. We first detail how PPI can be used in four separate common background strains of mice, detailing objective exclusion criteria including embedded test/retest reliability measures, introduce a non-parametric variable for within strain comparisons, and show 50% PPI determination measures. We then employ these measures to assess behavioral responses in quiet and background noise in mice (with their littermate controls) either lacking the MOC receptor subunit α9 nicotinic acetylcholine receptor [α9 nAChR (–/–)] or expressing an overactive receptor [Ld'T mutation in α9 nAChR KI], or lacking a LOC efferent neuropeptide, alpha calcitonin gene-related peptide [αCGRP (–/–)] (Elgoyhen et al., 1994; Lu et al., 1999; Maison et al., 2003a; Taranda et al., 2009). Because CGRP receptor formation matures from juvenile to adult ages, which is correlated with the increase observed in wave 1 amplitude of the auditory brainstem response (ABR), we also asked if a similar maturation in PPI responses can be observed between juvenile and adult (+/+) control and adult CGRP (–/–) animals (Dickerson et al., 2016).

## Materials and methods

### Animals

Two inbred strains of mice were obtained as breeder pairs from Jackson laboratories (Bar Harbor, ME) and male and female mice were bred at the University of Rochester vivarium: C57BL6/J JAX# 664; *N* = 14 (7M/7F), CBA/CaJ JAX #654; *N* = 12 (6M/6F). The inbred strain 129S6 SvEvTac mouse line was obtained from Taconic Laboratories (cat. 129SVE), *N* = 13 (7M/6F). We then generated the F1 hybrid C57BL6/J x 129S6 SvEvTac mice *N* = 12 (6M/6F). The [α9 nAChR (–/–)] and their littermate controls were generated from heterozygous matings of JAX #5696 CBACaJ;129S-*Chrna9*^*tm*1*Bedv*^/J, and *N* = 12 (6M/6F) of both (–/–) and (+/+) were tested. Mice expressing an overactive receptor [Ld'T mutation in α9 nAChR KI] were a gift from Dr. Douglas Vetter (Taranda et al., 2009). These mice were generated and maintained on a FVB/NJ background and bred as heterozygotes for transgenic mice expressing the Ld'T mutation and wildtype littermate controls, with *N* = 12 (6M/6F) mice in each group. And finally, αCGRP (–/–) mice (on a 129SvEv background), originally generated by Dr. Ron Emeson's laboratory (Lu et al., 1999) with an active breeding colony at the University of Rochester (Dickerson et al., 2016), were bred as heterozygotes to generate αCGRP (–/–) null and (+/+) wildtype littermates. Again *N* = 12 (6M/6F) αCGRP (–/–) and (+/+) mice were tested. All mice were group-housed in a controlled constant climate and 12/12 h normal light/dark cycle, with food and water available *ad libitum*. Testing was performed in the daylight hours. The University of Rochester Committee on Animal Resources (UCAR) approved all procedures, which were in accord with NIH guidelines, USPHS regulations, AALAC, and the Federal Animal Welfare Act.

### Apparatus

Experiments were conducted within a sound-attenuating room (IAC, Bronx, NY) with Sonex foam lining the walls. One mouse was tested at a time while confined in an aluminum wire cage, 5 cm wide, 7 cm long, and 4 cm high, having free sound penetration. The testing cage was oriented so that the mouse's head faced a TDT-ES2 electrostatic speaker (Prepulse Speaker) located 46 cm directly in front of the mouse's head. A second ES2 speaker (Masker Speaker) was positioned 7° to the left of the Prepulse Speaker, and this broadcast the masker when it was present. The cage was mounted on a 15-cm long pedestal that was bolted to a suspended acrylic platform to which an accelerometer was attached. The startle speaker was a Yamaha JA4281B compression tweeter that was suspended 15 cm above the mouse. The Startle Speaker and its supports, the pedestal and the acrylic shelf, and the table on which the apparatus was placed were all covered with echo absorbing foam or carpeting. Prior to testing all animals were pre-screened using both distortion-product otoacoustic emissions (DPOAEs) and click auditory brainstem responses (ABRs), and all animals passed these screens of cochlear function using methods previously described (Dickerson et al., 2016). Animals passed cochlear function testing if DPOAE amplitudes were greater than 6 dB SNR (DPOAE amplitude at least 6 dB above noise floor) for frequencies between 5 and 32 kHz, and ABR thresholds to click stimuli were less than 15 dB. Startle eliciting stimuli (ES) were 15 ms broad-band noise bursts (5 ms linear-gating, 50 kHz bandwidth, 120 dB SPL) digitally generated using a Tucker-Davis Technology (TDT, Alachua, FL) RP2.1 Real-time Processor, attenuated using a TDT PA5, then amplified with an Adcom (East Brunswick, NJ) GFA-535 II amplifier and broadcast from the Startle Speaker above the mouse. Prepulse and masker stimuli (similar bandwidth as startle eliciting stimuli (2–100 kHz) but at 60 dB SPL) were digitally generated using a second TDT-RP2.1 (100 kHz sample rate) and broadcast from the two ES2 speakers. The force of the startle reflex was transduced by the accelerometer and the voltage output sampled at 1 kHz by the first RP2.1. The startle response amplitude was the RMS of this output in the 100 ms period after the delivery of the startle stimulus. The experiment was controlled from a PC using a custom Matlab (The Mathworks, Inc) front-end. Sound levels were measured with a ¼″ microphone (Bruel and Kjær model 4135) using linear weighting connected to a measuring amplifier (Bruel and Kjær model 2610).

### Procedures

Each testing session began with the mouse being placed within the testing cage in the startle chamber for a 2-min acclimatization period, prior to delivery of stimuli. Each session had 11 presentations of each condition, these being block randomized, and the inter-trial interval was randomized between 15 and 25 s. The responses from the first block were not analyzed to avoid potential large responses on the first few trials. The duration of each session was 25–45 min, with each session separated by at least one rest day. All mice completed each of the six test sessions within 2 weeks of the initial testing session.

### Acoustic startle response (test day 1)

There was no prepulse in these sessions, which were designed to characterize the startle response of each mouse. There were 12 conditions in this session; startle stimuli were 80–130 dB SPL, and delivered in 5 dB steps either in silence or in a continuous background noise, which, when present, was on for the duration of the inter-trial interval and broadcast from the Masker Speaker.

### Gap detection (test day 2)

The prepulse was a 10 ms silent gap embedded in an otherwise continuous 50 kHz bandwidth white noise, delivered from the Prepulse Speaker. The level of the acoustic startle was 120 dB SPL. There were 6 conditions in this session; four prepulse conditions with interstimulus interval 10, 60, 100, and 300 ms, and two no-prepulse control conditions.

### Signal detection in quiet (test day 3/5)

The prepulse was a 40 ms duration broadband noise burst with 15 ms linear-gated rise-fall time, broadcast from the Prepulse Speaker. The interstimulus interval was 100 ms and the startle level was 120 dB SPL. There were 12 conditions in this session; prepulses 30–75 dB SPL in 5 dB steps, and two no-prepulse control conditions.

### Signal detection in noise (test day 4/6)

The prepulse was a 40 ms duration broadband noise burst with 15 ms linear-gated rise-fall time, broadcast from the Prepulse Speaker. A continuous broadband 60 dB SPL masker was played from the Masker Speaker for the duration of the session. The interstimulus interval was 100 ms and the startle level was 120 dB SPL. There were 12 conditions in this session; prepulses 48 to 75 dB SPL in 3 dB steps (−12 to +15 dB S/N), and two no-prepulse control conditions.

### Analysis

Startle test-retest reliability was calculated using bivariate correlation of the mean control startle response for the Signal in Quiet and Signal in Noise sessions, Test Days 3 & 5 and 4 & 6 respectively. Startle ratios were calculated for ASR sessions as the simple ratio of the mean response in the Masker condition to the mean response in the Quiet condition, for each level of the startle stimulus. PPI scores were calculated as a ratio of the each subject's mean startle response amplitude in the prestimulus condition (ASR_p_) compared with the no-prepulse control baseline (ASR_c_), PPI = 1 − [ASR_p_/ASR_c_]. PPI reliability was assessed using the Area-Under-the-ROC curve, A', calculated from the 20 control ASR trials per session and the 10 PPI trials for each condition, with first block excluded. Repeated-measures analyses of variance (ANOVA) were performed with SPSS v.16 (SPSS Inc, Chicago, IL). Graphical presentation of the data and supplemental ANOVAs and *t*-tests on specific stimulus conditions within- and between-subjects used GraphPad Prism software (version 5).

## Results

### Acoustic startle response (ASR)

**Figure 1:**
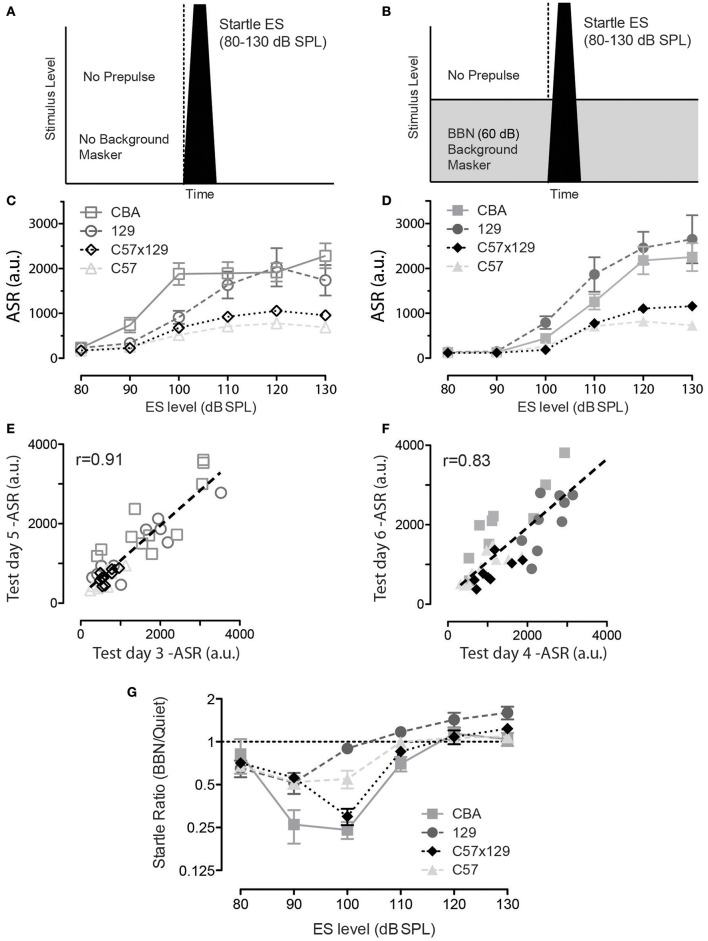
A robust acoustic startle response (ASR) is present in all mouse strains tested, yet varies by sound level, mouse strain, and background noise. **(A)** Schematic of startle stimulus presented in otherwise quiet background conditions. **(B)** Schematic of startle stimulus embedded in continuous 60 dB SPL broadband background noise. **(C)** For each of the four mouse strains tested, the ASR increases with sound level from 80 to 120 dB SPL with near maximal startle elicited in each strain by 120 dB SPL stimulus levels. **(D)** For each of the four mouse strains tested, at low startle levels, the ASR is reduced compared to when it is delivered in quiet, likely by sensory masking of the startle sound by the background noise; CBA *n* = 12 animals; 129 *n* = 12 animals; c57 × 120 *n* = 12 animals; c57 *n* = 12 animals. At higher sound levels the ASR increases. **(E,F)** There is high test/retest reliability for individual mice for both the ASR in quiet **(E)** and for the ASR in background noise **(F)**, while there may be variability within and between strains. **(G)** A new metric, the ASR ratio (BBN/quiet) was computed to compare the ASR in noise to the ASR quiet responses. Note that a startle level (ES) of at least 120 dB is needed to ensure and ASR ratio of 1.0 or higher. Error bars are SEMs.

This hearing in noise measure in the mouse model makes use of the ASR and the ability of prepulses to inhibit the ASR to assess the mouse's ability to detect prepulse signals presented in quiet or embedded in masking noise. Therefore, this measure will only be robust if an animal's ASR (without any PPI) is greater than the activity noise floor, and our first goal then was to verify if each of the mouse strains exhibited a robust ASR in quiet and in the presence of a 60 dB background noise. These two startle paradigms are shown in Figure 1A for ASR in quiet and in Figure 1B for ASR in background noise. Figure 1C shows ASR in quiet for the four strains tested, and each curve shows an increase in ASR with increasing loudness of the startle stimulus (ES). There is a difference in ASR by strain with C57 < C57 × 129 < 129 < CBA. Figure 1D shows ASR in the presence of background noise (60 dB BBN), and how this has modified the ASR functions. All mice exhibit a similar profile as in quiet (C57 < C57 × 129 < 129 < CBA), but again the magnitude varies by strain, C57 is the smallest, the hybrid is marginally higher, CBA and 129 are both large. Performing such a startle series in quiet and masking noise can also serve as an exclusion criteria as the startle response should increase as the ES level increases, and if this is not the case then the animal does not have a robust startle response and should be excluded. Strains exhibit differences in ASR in both quiet and in noise, yet all strains tested have a stable startle response for 120 dB SPL, and because of this 120 dB SPL is the startle amplitude level used for all future testing. As mentioned in methods, when PPI was assessed, there are 20 blocks with no prepulse, and because we tested PPI on two alternate days (test day 3 and 5 for quiet; test day 4 and 6 for masking BBN), we were able to show test/retest reliability of startle in both quiet and noisy backgrounds. Figures 1E,F show test-retest reliability of startle in the mice studied showing individual mice have reproducible startle responses. Reliability is high, *r* = 0.91, *p* < 0.001 (quiet), and 0.83, *p* < 0.001 (noise).

### Acoustic startle response ratio

However, as can be seen in Figure 1D, the presence of noise has reduced the size of the low level startle, especially at 90 and 100 dB. This is highlighted in Figure 1G, which shows a new metric, the ASR ratio, which is an animal's ASR measured in noise, divided by that animal's ASR in quiet. The CBA strain shows the most dramatic reduction in responding in noise, but the C57x129 hybrid is also strongly affected at 100 dB. Such variability suggests caution when using low-level startle in background noise, as reduced startle magnitude affects the signal-to-noise ratio of the response. At the startle stimulus 120 dB SPL the startle ratio is ~1.0 for all strains tested, as shown in Figure 1G, and that is the startle stimulus used for future PPI experiments.

### Gap detection

**Figure 2:**
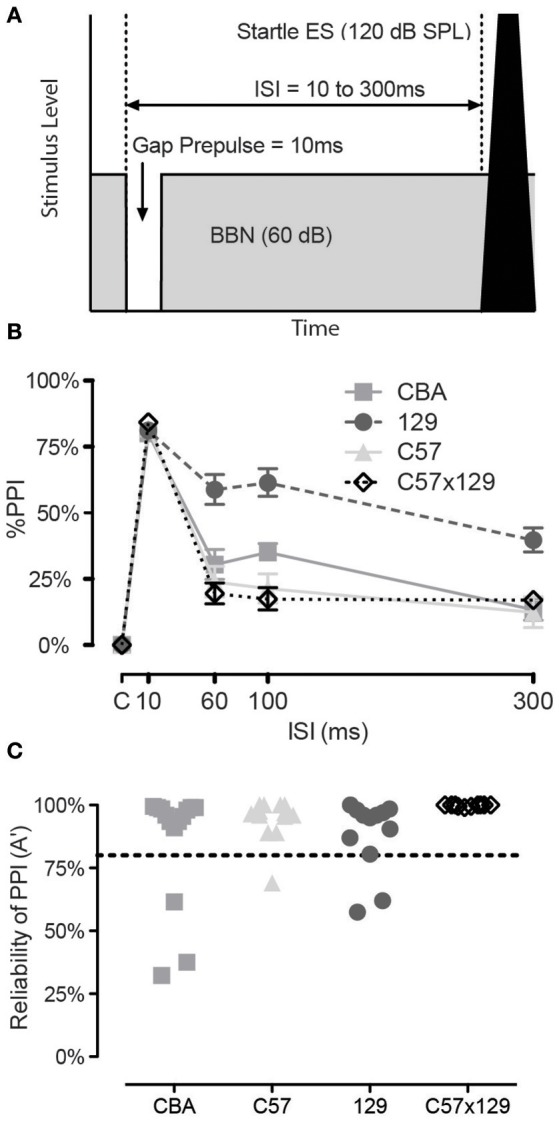
Prepulse inhibition for a gap in otherwise continuous background noise can be used for rejection criteria. **(A)** Schematic of the acoustic stimuli. The prepulse is a 10 ms silent gap that commences 10–300 ms prior to the startle stimulus. **(B)** The gap is a highly effective prepulse in all mice when presented at 10 ms lead-time before the ASR. Strains vary by how effective the prepulse is beyond 10 ms, though the response is stable between 60 and 100 ms. **(C)** Criterion for rejecting individual mice. If the non-parametric A′ measure was less than 80% for the 10 ms gap condition, the mouse would be rejected as an unreliable responder. Strains varied in the proportion of rejected animals. Error bars are SEMs.

In addition to evaluating ASR we wanted an independent exclusion measure to ensure mice could inhibit their ASR, yet did not want this exclusion measure to be part of our hearing in noise testing. We determined that pre-pulse inhibition (PPI) for a gap in otherwise continuous background noise could serve as a secondary exclusion test. Figure 2A shows a schematic of the acoustic stimuli, where the prepulse is a 10 ms silent gap that commences 10–300 ms prior to the 120 dB startle stimulus, with an inter-stimulus interval of 10 ms being the most effective for eliciting pre-pulse inhibition (PPI) as shown in Figure 2B. We determined a criterion for rejecting non-responding animals by using a non-parametric A′ measure, excluding if the response was less than 80% for the 10 ms gap condition, as shown in Figure 2C. As can be observed, the various mouse strains varied as to the proportion of rejected animals with CBA having the most unreliable responders and C57x129 hybrid strain with no non-responders. Interestingly, these excluded mice were the same animals that had the weakest growth to increasing startle levels, highlighting that the growth of the startle response may also be good exclusion criteria, especially if gap detection will be later assessed for temporal processing abilities.

### Signal detection in quiet/noise in background strains

**Figure 3:**
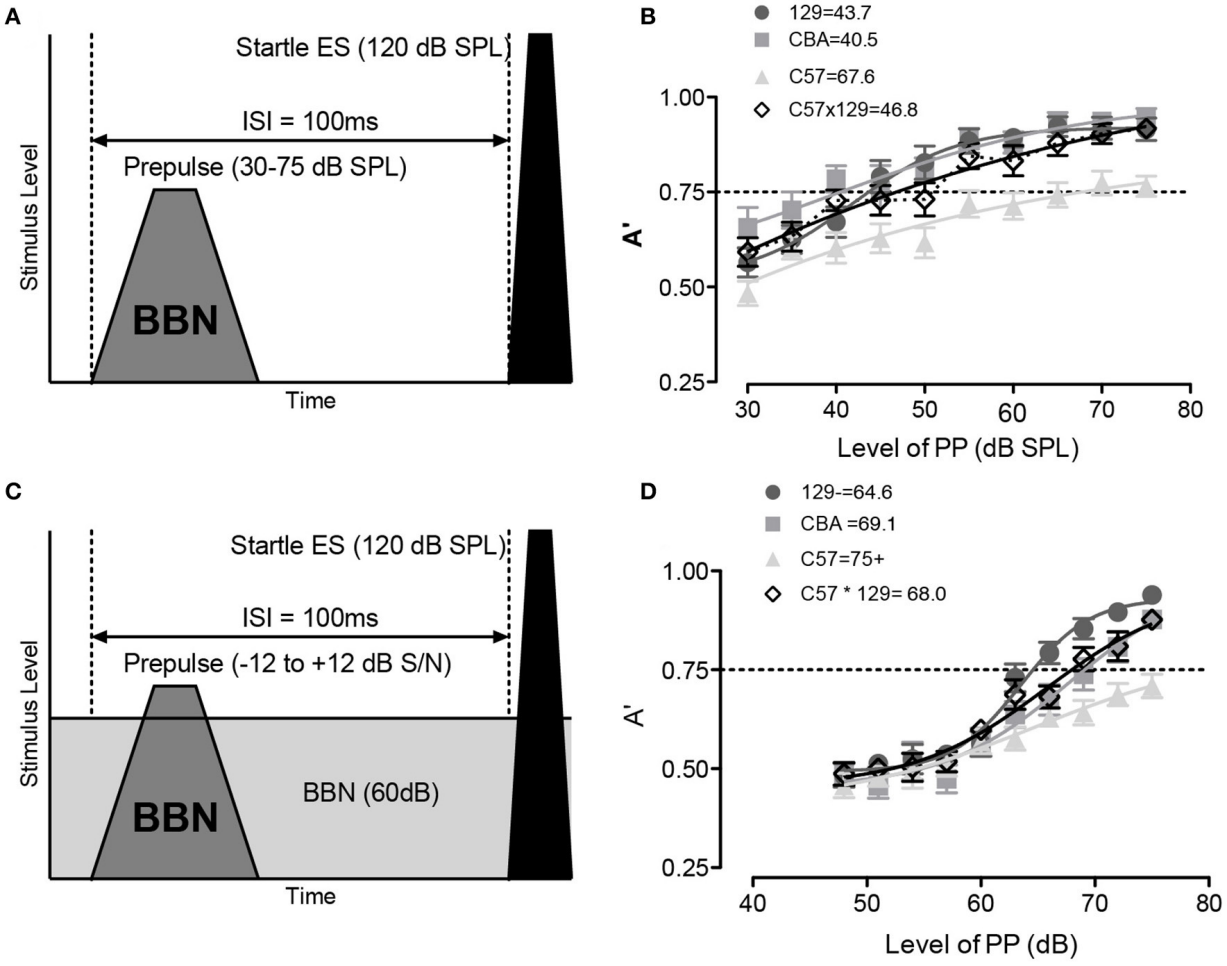
When brief prepulses are detected in quiet and masking noise, they can be used to inhibit the ASR [i.e., prepulse inhibition (PPI)]. PPI can be used then to assess the mouse's ability to detect prepulse signals presented in quiet or embedded in masking noise. **(A)** Schematic of prepulse and acoustic startle stimulus. The prepulse was a broad band noise 2–100 kHz (BBN), the acoustic startle stimulus was always a BBN delivered at 120 dB. **(B)** Detection of the broadband (BBN) prepulse was robust in all strains, and increased with level, with A′ = 0.5 equal to no difference between no prepulse and prepulse (i.e., not detectable), and 50% PPI threshold defined as sound level with A′ = 0.75, and the numbers next to strain labels indicate the prepulse level (dB SPL) at which this is reached. C57 mice were unusual in having a low asymptotic A′ to PPI compared to the other strains. **(C)** Schematic of prepulse and startle stimulus in background masking noise (2–100 kHz@ 60 dB). **(D)** Detection of the broadband prepulse against the masking noise was again robust in all strains. Error bars are SEMs.

Once the non-responding animals were culled from the test animals, we assayed the animals' ability to use a brief prepulse of broadband noise (BBN, 2–100 kHz) to inhibit the acoustic startle (120 dB BBN). These tests were performed in both quiet (schematized in Figure 3A) and in the presence of background noise (schematized in Figure 3C) and each animal's inhibition of the ASR was tested over 20 times in each test condition. A non-parametric measure A′ was defined as 1.0 if the PPI is completely separable from the no-prepulse control condition and varied to 0.50 which is defined as not separable from the control condition. Converting the ASR values to A′ measures allowed for normalization across animal and strain as we had observed strain differences in the ASR raw magnitude. As shown in Figure 3B, all strains were able to inhibit ASR to at least 50% (A′ = 0.75) with BBN prepulses. When background noise was added to the prepulse detection, a louder prepulse stimulus was needed to cause the same inhibition of the ASR, yet all strains were able to inhibit their reflex at the loudest prepulse tested (Figure 3D). As can be observed, the 50% PPI level (A′ = 0.75) did vary by background strain, highlighting importance of using littermate controls when testing genetically modified animals.

### Signal detection in quiet/noise in mice lacking αCGRP (an LOC neurotransmitter)

**Figure 4:**
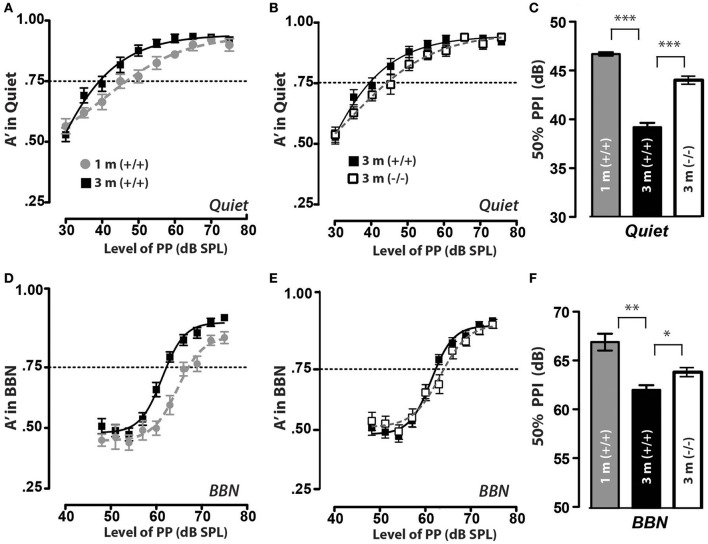
Prepulse inhibition improves during juvenile to adult maturation with the LOC neuropeptide CGRP. **(A)** In quiet, CGRP (+/+) animals of both ages can inhibit their acoustic startle with a prepulse at 80 dB, and did not inhibit their startle with a prepulse at 30 dB (edges of psychometric function). However, the threshold for inhibiting their startle response 50% of presentations (A′ = 0.75) is higher at 1 month (1 m) and drops by 3 m of age. **(B)** Similarly, in quiet, CGRP (–/–) animals can inhibit their acoustic startle yet the threshold for inhibiting their startle response 50% of the time (A′ = 0.75) is higher at for CGRP null (–/–) animals when compared to CGRP wildtype control (+/+) animals tested at 3 months of age. **(C)** Mean PPI thresholds (defined as prepulse level at A′ = 0.75) and SEM values are shown for both CGRP (+/+) solid bars and CGRP null (–/–) animals (open bars), and each group contained 12 mice. There was a significant threshold increase for CGRP (+/+) animals between 1 and 3 m (^***^p < 0.001). **(D,E)** When prepulse inhibition of the acoustic startle reflex was tested in 60 dB background noise (BBN), CGRP (+/+) and CGRP (–/–) mice of both ages were able to inhibit their startle reflex when tested with a prepulse of 80 dB, but did not inhibit their startle with a prepulse at 50 dB; yet similar to the quiet condition, the threshold for 1 month animals was higher than for the same animals at 3 m. **(F)** There was a significant decrease in PPI thresholds in BBN in CGRP (+/+) between 1 and 3 m (^**^p < 0.01), and a less significant increase in PPI thresholds in CGRP null (–/–) animal's thresholds with maturation (^*^p < 0.05).

As mentioned earlier, we wanted to determine if the difference in suprathreshold cochlear nerve responses between juvenile and adult mice was reflected in differences in the animals' PPI responses in quiet and in noisy backgrounds (Dickerson et al., 2016). We tested PPI in the same control mice [CGRP (+/+) littermate controls] as juveniles at 1 month of age (1 m) and then again at 3 months as adult mice (3 m), using similar exclusion criteria as described earlier. The background strain for the αCGRP (–/–) is a 129 SvEvTac mouse line which are very good responders for PPI and no mice were excluded and for completeness, we performed PPI in quiet and background noise 2x each (testing 1x/day) at 1 m and then later at 3 m of age. We also assessed PPI responses in CGRP (–/–) mice at 3 months, as we have previously shown that the loss of CGRP (–/–) reduced cochlear nerve activity (Maison et al., 2003a). The CGRP (+/+) juvenile mice could detect the prepulse and inhibit the acoustic startle with 50% PPI at 46 dB (0.242 SEM) when the prepulse was delivered in a quiet background, as shown in Figures 4A–C (gray circles/bars). When the prepulse was delivered in a background noise, the PPI threshold in juvenile mice was 67 dB (0.248 SEM), as shown in Figures 4D–F (gray circles/bars). However, when these same animals were allowed to age and were then retested 2 months later, their 50% PPI responses decreased by 7 dB (0.240 SEM) in quiet and 4 dB (0.141 SEM) in a noisy background (black squares/bar in Figure 4), suggesting that the increase in suprathreshold response in the cochlear nerve that occurs during maturation results in behavioral changes that are observed in quiet and broadband noise (BBN) settings. We did not observe any differences between male and female mice in PPI thresholds for both quiet and BBN conditions.

### Signal detection in quiet/noise in mice lacking or expressing an overactive α9nAChR (an MOC receptor)

**Figure 5:**
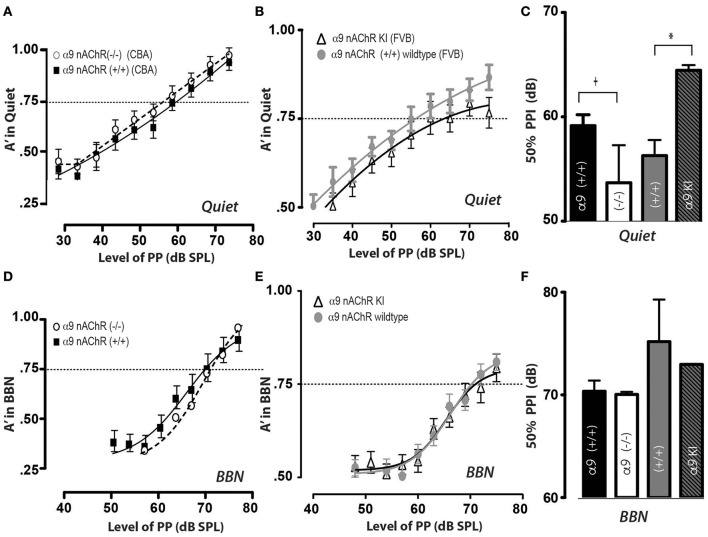
Prepulse inhibition thresholds in quiet are modified in opposite directions by the loss (–/–) or increased activity Ld'T (KI) of α9 nAChRs, yet responses do not differ significantly in background noise. In quiet, **(A)** α9 nAChRs (–/–) or **(B)** Ld'T KI animals can inhibit their acoustic startle with a prepulse at 80 dB, and did not inhibit their startle with a prepulse at 30 dB (edges of psychometric function). However, the threshold for inhibiting their startle response 50% of the time (A′ = 0.75) is higher for α9 (–/–) null animals **(A)** and lower for α9 KI animals **(B) (C)** Mean PPI thresholds (defined as prepulse level at A′ = 0.75) and SEM values are shown for **(A)** α9 nAChRs (–/–) or B) Ld'T KI, and each of the groups contained 12 mice. There was a significant threshold increase for α9 nAChR KI animals when compared to littermate controls) and there was a trend for α9 nAChRs (–/–) to have lower thresholds in quiet (*t*, trend; ^*^p < 0.05). **(D,E)** When prepulse inhibition of the acoustic startle reflex was tested in 60 dB background noise (BBN), α9 nAChRs (–/–) or Ld'T KI mice were able to inhibit their startle reflex when tested with a prepulse of 80 dB, but did not inhibit their startle with a prepulse at 50 dB; yet unlike the quiet condition, there was no significant difference between **(D)** α9 nAChR (–/–) or **(E)** Ld'T KI mice in their response, as shown in bar graph form with means and SEMs in **(F)**.

And finally, we were interested in determining if the MOC efferent pathway mediated by α9 nAChR responses plays a role in PPI responses in quiet and in background noise, as the MOC pathway has been shown to be necessary for sound protection in guinea pigs and rabbits (Luebke and Foster, 2002; Luebke et al., 2002, 2014; Maison et al., 2002). We have examined PPI abilities in mice (with their littermate controls) either lacking the MOC receptor subunit α9 nicotinic acetylcholine receptor [α9 nAChR (–/–) null] that have been backcrossed to the CBA strain, (Elgoyhen et al., 1994) or mice expressing an overactive receptor [Ld'T mutation in α9 nAChR KI] that are in the FVB strain (Taranda et al., 2009). Using our exclusion criteria, two of the α9 nAChR controls were excluded, and one mouse of the overactive nAChR receptor was excluded. We found that α9 nAChR (–/–) null mice show a trend toward reduced 50% PPI responses in quiet (shown in Figures 5A,C) when compared to littermate controls [WT (+/+) 59 dB, 1.05 SEM; (–/–) 52.7 dB, 3.6 SEM, *p* = 0. 282]. We also found that the mice carrying the [Ld'T mutation in α9 nAChR KI] exhibit a significant increase in their 50% PPI responses in quiet, as shown in Figures 5B,C [(+/+) WT 56 dB, 1.5 SEM; KI 64.5 dB, 0.05 SEM, *p* = 0.035]. These changes most likely reflect the rightward shift in the rate-level function when MOC efferents are stimulated (Winslow and Sachs, 1987, 1988; Sachs et al., 1989). Interestingly, when PPI was tested in the presence of background noise there were no significant differences between 50% PPI responses between α9 nAChR (–/–) mice and their littermate controls (Figures 5D,F; *p* = 0.80) nor between Ld'T mutation in α9 nAChR KI animals and their FVB littermate controls (*p* = 0.64), as shown in Figures 5E,F. Again, as we had found for the CGRP (–/–) and (+/+) mice, we did not observe any differences between male and female mice in 50% PPI responses for any of the conditions examined.

## Discussion

We have described a hearing-in-noise test that can be used in the mouse model, which makes use of the ASR and the ability of prepulses to inhibit the ASR to assess the mouse's ability to detect prepulse signals presented in quiet or embedded in masking noise. The ASR was measured in response to brief 80–130 dB SPL noise bursts, delivered in quiet or in the presence of a 60 dB SPL background noise, and determined a startle threshold and optimum level for maximal ASR. We then delivered prepulses at 48–72 dB SPL in either quiet or 60 dB SPL broadband noise masker, and assessed PPI.

Because the background strains of many transgenic mice lines differ, we studied this hearing-in-noise behavior across four inbred mouse strains (CBA/CaJ, C57BL6/J, 129SvEv, C57BL6/J × 129SvEv). We have determined that this test is robust and exhibited high test/retest reliability in all mouse strains tested, and that there is a masked threshold, below which there is no PPI and above which PPI grows with increasing prepulse level. We also have used these techniques on mice lacking MOC receptor [α9 nAChR (–/–) null] or on mice with an overactive MOC receptor [Ld'T mutation in α9 nAChR KI] and mice lacking a LOC neurotransmitter (CGRP) at juvenile and adult ages, and these PPI measures can reliability be used to assess behavioral responses in quiet and background noise.

### Factors contributing to PPI measure robustness

This described method of detecting hearing-in-noise differences in the mouse model has many features that contribute to the measure's robustness. This RMA-based testing of hearing in noise ability is relatively quick to administer and does not require training. However, we limited our test sessions to <45 min testing per day per mouse, and found extremely high test/retest reliability of ASR responses (*r* = 0.91 or *r* = 0.83, *p* < 0. 001), and believe if testing is longer than 45 min, fatigue and/or habituation could become a factor as discussed earlier (Lobarinas et al., 2013; Lauer et al., 2017). We have also made use of the startle series, the ASR ratio, and the well-established gap-detection paradigm (e.g., Turner and Parrish, 2008; Turner and Larsen, 2016), to serve as an exclusion criteria. The ASR ratio (startle in background/startle in quiet) should be near 1.0, which required a startle stimulus level of at least 120 dB SPL, which is higher than others have used for PPI testing and could then have resulted in difficult to interpret masked thresholds (Hickox and Liberman, 2014). While both a broadband (BBN) prepulse and an octave band (OBN) prepulse (data not shown) were capable of eliciting PPI in all mouse strains, the broadband prepulse was a much more effective elicitor of PPI in both quiet and noisy conditions and has been used throughout these studies.

We have also developed a new metric, acoustic startle ratio (ASR), which is the ratio of an animal's acoustic startle magnitude in broadband noise (BBN) to its ASR magnitude in quiet. We determined that a startle stimulus of 120 dB SPL is needed to yield an ASR ratio close to 1.0 for all mouse strains tested. We have also presented an objective method to determine behavioral PPI thresholds in quiet and in noise and have found these measures to be robust in all mouse strains tested when broadband prepulses are used. While this method is robust for all strains tested, we did find strain differences, pointing to the importance of using similar littermate-background strains when this method is used in genetically altered animals (as is shown in Figures 4, 5 for LOC and MOC genetically altered animals). Moreover, as this hearing-in-noise test is based on the ability of animals to inhibit their acoustic startle reflex when they detect a prepulse delivered in quiet or background noise, this assay cannot be used in mice with motor deficits as their ASR may vary due to the motor weakness and not due to auditory cues. Other limitations of this method are discussed in this recent review by Lauer (Lauer et al., 2017).

In addition to increased noise and ototoxic susceptibility, we determined juvenile mice have higher PPI thresholds in quiet and background noise. Dickerson et al. noted that suprathreshold cochlear nerve activity increased by 30% between juvenile (1 m) and adult mice (3 m) (Dickerson et al., 2016), and these studies suggest that such an increase in cochlear nerve activity (30%) could translate into a 4–5 dB improvement in 50% PPI responses, suggesting that even moderate losses of sound-evoked activity in the auditory nerve (as has been documented for temporary threshold shifts to noise exposures) may have more severe consequences on suprathreshold behavioral responses (Kujawa and Liberman, 2009, 2015; Furman et al., 2013). Interestingly, Longenecker and Lobarinas have both found PPI thresholds (assessed in quiet) were similar to ABR wave 1 amplitude losses when animals were noise-exposed; yet found PPI thresholds remained stable, whereas ABR thresholds recovered (Longenecker and Galazyuk, 2016; Longenecker et al., 2016; Lobarinas et al., 2017). Moreover, as PPI testing does not involve anesthesia, efferent feedback is maximized (Maison et al., 2012; Aedo et al., 2015).

### Dual roles for olivocochlear neurotransmission

In addition to the hearing-in-noise PPI test development with exclusion criteria, the main finding is that both MOC and LOC efferent feedback are needed for efficient PPI behavioral responses. The MOC reflex has been studied almost exclusively by measuring changes in otoacoustic emissions (Guinan, 2014) yet this ignores the LOC system contributions, and can underestimate effect of sound-evoked efferent feedback (Lichtenhan et al., 2016). Because the MOC projection is myelinated and the LOC projection is not, stimulation of the IVth floor of the ventricle also predominately activates the MOC pathway. However, it is possible to selectively disrupt the LOC pathway using a neurotoxin and Le Prell et al. (2014a) found that disruption of LOC neurons depressed spontaneous cochlear nerve activity. When the CGRP component of the LOC system was eliminated, the loss of CGRP caused reduced cochlear nerve activity and resulted in increased 50% PPI thresholds in quiet and in background noise.

With MOC disruption [α9 nAChR (–/–) null] or an overactive receptor [Ld'T mutation in α9 nAChR KI] we found that the 50% PPI thresholds were modified in opposite directions (i.e., [α9 nAChR (–/–) null] animals exhibited lower 50% PPI thresholds in quiet and [Ld'T mutation in α9 nAChR KI] animals exhibited increased 50% thresholds in quiet. These 50% PPI response changes with the MOC pathway silenced or over-activated, are similar to what is predicted by the rate-level functions of auditory unit recordings with and without MOC stimulation (Winslow and Sachs, 1987, 1988; Sachs et al., 1989). We had expected to observe a PPI threshold shift in background noise but did not observe any significant differences, yet this lack of significance could be due to the higher variance observed in the α9 nAChR (–/–) animals, as has been observed in other studies using this mouse line (Lauer, 2017). We are not sure if higher order pathways could have compensated for genetically altered MOC feedback (Salvi et al., 2000, 2016; Auerbach et al., 2014), yet if there was higher-order compensation, this compensation did not fully negate changes observed in the CGRP (–/–) animals. Another alternative is that the MOC and LOC pathways, and all neurotransmitters and receptors involved work in tandem to improve hearing in noisy backgrounds. (Maison et al., 2003b, 2013). These experiments pave the way for behavioral hearing-in-noise assessments with exclusion criteria in mice with other efferent-feedback deficits, or mouse models with presumed auditory filtering deficits.

## Author contributions

Both AL and PA conceived and designed experiments, and participated in the acquisition, analysis, and interpretation of the experimental findings, wrote and revised the manuscript, gave final approval and also accountable to all aspects of the work.

### Conflict of interest statement

The authors declare that the research was conducted in the absence of any commercial or financial relationships that could be construed as a potential conflict of interest.
